# The development of a capability wellbeing measure in economic evaluation for children and young people aged 11-15

**DOI:** 10.1016/j.socscimed.2024.117311

**Published:** 2024-09-08

**Authors:** Samantha Husbands, Paul Mark Mitchell, Philip Kinghorn, Sarah Byford, Katie Breheny, Cara Bailey, Paul Anand, Tim J. Peters, Isabella Floredin, Joanna Coast

**Affiliations:** a Health Economics & Health Policy (HEHP@Bristol), Population Health Sciences, Bristol Medical School, University of Bristol, Bristol, BS8 1NU, UK; b Health Economics Unit, Institute of Applied Health Research, University of Birmingham, Birmingham, B15 2TT, UK; c King’s Health Economics, Institute of Psychiatry, Psychology & Neuroscience, King’s College London, London, SE5 8AF, UK; d School of Nursing, Institute of Clinical Sciences, College of Medical and Dental Sciences, University of Birmingham, Birmingham, B15 2TT, UK; e Economics, The Open University, Department of Social Policy and Intervention, Oxford University, Oxford, UK; f Population Health Sciences, Bristol Medical School, and Bristol Dental School, University of Bristol, Bristol, BS1 2LY, UK

**Keywords:** Capability approach, ICECAP measures, Children and young people, Economic evaluation, Qualitative methods

## Abstract

The capability approach provides a broad evaluative space for making funding decisions for health and care interventions, with capability wellbeing as the outcome of value. A range of capability measures have been developed for the economic evaluation of health and care interventions for adults. However, such measures have not been previously developed for children and young people for this purpose and may be valuable. This study aimed to identify important capabilities for children and young people aged 11–15, and to develop these into attributes for an economic measure that can inform funding decisions. Thirty-three qualitative in-depth interviews were undertaken with children and young people aged 11–15 (n = 19) and parents (n = 14) in urban and rural areas of England between September 2019 and November 2021. Purposeful maximum variation sampling ensured representation from different backgrounds. Children and young people were asked to think of things important to them and place these on sticky notes around a drawing/photograph of themselves; the interview asked them about these important things. Parents were asked to identify factors that enhanced and negatively impacted their child’s quality of life. Analysis using constant comparison facilitated exploration of similarities and differences in important capabilities. A second phase of semi-structured interviews with children and young people (n = 15) explored how these attributes should be expressed in a meaningful way. Eight overarching capability wellbeing themes were identified, with some variation across children and young people, and parent groups: **Fun and enjoyment; Learning and experiencing; Attachment; Emotional security and support; Achievement; Identity and choice; Physical safety; Aspiration**. Potentially, this information will help to provide an alternative approach to the measurement of benefits to children and young people for economic evaluation of health and care interventions, one that will be better able to capture benefits associated with interventions to improve the social determinants of health.

## 1 Introduction

There has been increasing interest in developing methods for economic evaluation in children and young people (CYP) in recent years, with a number of reviews published exploring the current state of the art across both measurement and valuation issues (Bailey et al., 2022; Chen and Ratcliffe, 2015; Hill et al., 2020; Kwon et al., 2018, 2022; Rowen et al., 2020) and the funding of new research programmes (see, for example, the funding of two major programmes of work in this area in Australia: QUOKKA https://www.quokkaresearchprogram.org/and TORCH https://www.torchstudy.com.au/). Most work in this area has retained the traditional focus of measuring health-related quality of life, as has been the most usual practice in adult economic evaluation. There are policy moves in health and social care, however, that suggest that a broader approach might be helpful. In England, for example, a recent restructure of health service decision making has replaced health decision making bodies at local level with Integrated Care Systems (ICSs) (“Health and Care Act,” 2022). ICSs have responsibilities for improving population health and tackling inequalities, enhancing value for money and helping the National Health Service (NHS) to support broader social and economic development. The combination of these objectives gives ICSs a greater concern to address social determinants of health in their decision making. Policies to address issues such as cost of living, access to energy, deprivation and mental wellbeing are starting to transform ways in which local health and care services work. While contributing to population health outcomes, these policies have much wider impacts across all of society, suggesting that broader wellbeing outcomes are needed to capture the full impact of interventions. This is particularly important when thinking about economic evaluation in CYP, where many of these policies around the social determinants of health are focused (with, for example, the recent Hewitt review noting that “Giving every child the best start in life … is crucial to reducing health inequalities across the life course.” (Hewitt (2023), p.25)).

Use of the capability approach within health economics is now relatively established (Helter et al., 2019), with the measures accepted for use in decision making, particularly around adult social and long-term care (National Insitute for Health and Care Excellence, 2023; Zorginstituut Nederland, 2016). The approach advocates a shift in the evaluative space to one that focuses on the things that people are able to do and be in their lives: their capabilities (Sen, 1993). Application of the approach to CYP, however, raises a number of challenges (Mitchell et al., 2021). Early work in the area from the key original architects of the approach, Sen and Nussbaum, paid little attention to CYP given that CYP tend to be constrained in making their own decisions (Mitchell et al., 2021) either cognitively or legally, or both. Later work, however, has considered the capability approach in CYP in relation to ‘evolving capabilities’ (Ballet et al., 2011) and the ‘capability to aspire’ (seen as being instrumental in providing the basis for future opportunities to flourish and particularly relevant to the issue of social determinants of health) (Hart, 2014), and there has been some work with CYP around relevant dimensions for capability measurement regarding international child rights (Biggeri et al., 2006).

The development of capability approaches in health economics has taken two routes: capturing these capabilities through participatory approaches as advocated by Sen (for example, the ICECAP measures (Al-Janabi et al., 2012; Grewal et al., 2006; Sutton and Coast, 2014)), or drawing on Nussbaum’s ten central human capabilities (for example, the OxCAP measures (Lorgelly et al., 2015)). The participatory approach used in developing the ICECAP measures (Al-Janabi et al., 2012; Grewal et al., 2006; Sutton and Coast, 2014) fits well with increased use of co-production within health and social care (Rycroft-Malone et al., 2016). There are practical issues in developing capability measures for CYP with a participatory approach, however, which may require both innovative approaches (Husbands et al., 2020) and involving those beyond the child or young person. Given the rapid developmental trajectory of CYP, there are also issues around capturing capabilities that are sufficiently homogeneous within groups, yet sufficiently different between groups to justify differences in the measure. A combination of age and development often provides a guide as to where to make these splits between one measure and another; from a capability perspective, CYP capabilities may also be affected by the social and institutional contexts in which CYP live, which may also influence the capabilities that are expected or available to them.

This paper explores the capabilities that are important to CYP aged 11–15 years (secondary school age in the UK; mainly equivalent to lower secondary education on the International Standard Classification of Education (UNESCO Institute for Statistics, 2012)), both in terms of identifying the core concepts of importance, and determining how these should be expressed in a meaningful way, to generate an ICECAP measure for this age group. Ultimately, these meaningfully expressed concepts of capability wellbeing can be used in evaluations that will inform decision making around the use and funding of health and care interventions for children and young people in this age group.

## 2 Methods

### 2.1 Study design

Qualitative research with CYP aged 11 to 15 and their parents/guardians was undertaken to understand the important aspects of capability wellbeing that should be included as the conceptual attributes in a capability measure for CYP (the ICECAP-CYP:11-15 measure). The theoretical orientation for the work drew on both grounded theory approaches (Strauss and Corbin, 1990) and the application of an economic lens (Coast, 2017) related to the capabilities approach (Sen, 1993). The study design involved two phases of research: Phase one used in-depth interviews with CYP and parents to determine what was important to CYP and to generate conceptual attributes, and Phase two involved semi-structured interviews with CYP only, to check the coverage of the attributes and to develop meaningful wording for the attributes within the measure and meaningful presentation of these attributes. Parents’ views were included alongside those of CYP in generating the conceptual attributes, because CYP of this age are legally constrained in making their own decisions, with decision making generally the responsibility of parents. The work was part of a larger project focusing on CYP from 0 to 18 and covering broader issues around capability measurement in this group, including issues such as well-becoming (Husbands et al., 2024). Ethical approval for the study was gained from the University of Bristol Faculty of Health Sciences Research Ethics Committee (reference 77121) and a full protocol for the work is available (Husbands et al, 2022).

### 2.2 Sampling and recruitment

#### 2.2.1 Phase one

A purposeful, maximum variation approach was taken to sample CYP and parents/guardians from a broad range of backgrounds. CYP sampled included those from different age groups, socioeconomic backgrounds (determined using the English indices of deprivation 2019) (Noble et al., 2019), ethnicity, gender, household composition (including one and two-parent families) and those with and without health conditions. CYP could take part in the study with or without parents taking part, subject to parental consent (see below), but parents could only take part if their child took part. A gradual selection sampling approach was taken, with iterative decisions about who to approach next based on perspectives currently ‘missing’ from the research (Flick, 2009). Snowball sampling was used to approach and recruit siblings with the permission of the child and parent/guardian (Kuper et al., 2008).

CYP and their parents/guardians were recruited using multiple approaches. Prior to the start of the COVID-19 pandemic, recruitment was through schools and charitable organisations in the South-West of England. Recruitment was halted at the start of the COVID-19 pandemic. From late 2020, parents/guardians and CYP were recruited online from across England using the Facebook ‘targeted ads feature’ because of difficulties with recruiting via gatekeepers in organisations due to pandemic-related closures. Recruitment materials were developed for parents/guardians and were adapted for CYP to aid understanding. The materials were shared with a Young People’s Advisory Group and adjusted based on their insights. A pre-interview questionnaire allowed the research team to monitor the personal characteristics of those being recruited, to ensure all important demographics were represented.

#### 2.2.2 Phase two

In Phase two, informants who took part in Phase one were recontacted and asked to take part in this next phase. New informants were recruited using the Facebook targeted ads feature in a similar manner to that used in Phase one.

### 2.3 Data collection

#### 2.3.1 Phase one

In-depth interviews (Bryman, 2004) with CYP and parents/guardians were used to explore in detail what is important to the wellbeing of CYP, to generate the conceptual attributes for the measure. Interviews prior to COVID-19 were face-to-face in the family home or the organisation which facilitated recruitment. Following the onset of COVID-19, all interviews were conducted online, using Microsoft Teams. The presence of parents/guardians during interviews varied. For ethical reasons, CYP and parents were invited to mutually decide whether parents would stay or leave the room/Teams meeting. Parents who stayed were asked to not answer on the child’s behalf. Interviews were conducted by SH.

Considerable reflection was given to addressing power imbalances between adult researchers and CYP participants in the research. As a result, in all CYP interviews, a hierarchical mapping activity (Antonucci, 1986) was used. CYP were asked to think of things that were important to them and place these on sticky notes around a drawing/photograph of themselves, with the things most important to them closer to the drawing/photograph and those considered less important placed further away. This aimed to engage CYP, generate rich data and rebalance power relations between CYP and adult researchers by allowing CYP greater control over the discussion and how it took place (Barker and Weller, 2003; Punch, 2002). The activity was followed by open-ended interview questions asking why the CYP had recorded particular items as important to them, what it was about an item that made it important and why they had some items as more/less important than others. A separate topic guide was generated for parent interviews. Parents were asked questions on what they thought was important to their child’s quality of life and why, including how they thought it could be improved (see Supplementary material for topic guides).

#### 2.3.2 Phase 2

Single semi-structured one-to-one interviews with CYP were conducted to check the coverage of the attributes developed in Phase 1 and to generate language for the measure attributes and questions which could be easily interpreted by CYP aged 11–15 years. All Phase 2 interviews took place online using Microsoft Teams.

CYP were asked what certain words meant to them and were asked to explain their interpretations, to check that they understood the wording and that this matched the intended meaning of the developed attributes. Initially, interviews took options for wording directly from CYP interviews in Phase 1, to maintain the sorts of words used by CYP to feed into measure items (FDA, 2009; Matza et al., 2013). Interviews were undertaken in rounds to allow the wording to be updated and then tested in the next round. In the final round, a thinkaloud format was used (Ericsson and Simon, 1993) in which the final wording for the attributes was presented and CYP were asked to think aloud how they would see the attributes as applying to them (see supplementary material for an example topic guide from round 3).

The visual presentation of the attributes was also discussed with CYP. A number of visual presentations were tried, and CYPs were asked their views on these different formats.

### 2.4 Consent and assent processes

Tailored study information sheets were created for CYP and parents. All parents/guardians were asked to provide informed written consent for their child and for their own involvement. CYP were asked to assent to taking part to ensure that they were happy to participate and allow them an opportunity to ask questions (Morrow and Richards, 1996). Informants provided separate consent for each phase.

### 2.5 Data analysis

#### 2.5.1 Phase 1

Interviews were audio recorded and transcribed verbatim. They were conducted and analysed in batches of four to six to allow interview topic guides to be updated iteratively. Analysis used constant comparison (Strauss and Corbin, 1990), which involved continually comparing the meaning of new to existing data and later to emerging categories. Interview transcripts were coded line-by-line in NVIVO and the coding schedule was iteratively updated. Coding became more hierarchical as analysis progressed, with higher level categories linking lower-level codes to indicate the concepts most important to CYP’s wellbeing. Separate codes and coding schedules were generated for CYP and parents.

Analytic accounts were developed for each ‘batch’ of interviews to compare and contrast responses of CYP and parents separately (Coast and Jackson, 2017), paying attention to any experiences that did not fit with existing concepts (Mays and Pope, 2000). Analysis was conducted by SH, with regular review and discussion of coding and accounts between SH and JC. Emerging findings were regularly shared with the wider research team and advisory group to check interpretations. Data collection and analysis continued until data saturation, considered to be the point at which the conceptual attributes were fully developed, and no new codes or categories were emerging from any of the CYP or parent interviews to suggest that important aspects of CYP’s wellbeing had not already been captured (Morse, 1995; Saunders et al., 2018). As interviews for the broader study (ages 0–18) were conducted in parallel, the question of where to set the age boundaries for instruments was considered early across all analysis.

#### 2.5.2 Phase 2

Interviews in Phase 2 were analysed by round using methods of constant comparison to compare CYPs’ feedback on different options for wording and presentation. Analytic accounts were shared between, and discussed by, SH, PM, and JC, to decide which options for wording and presentation should be carried forward for testing in the next round. The measure was finalised when consecutive interviews had (i) not raised any issues with language or presentation, (ii) the discussion by the CYP in interviews suggested that the final wording represented what was included in the Phase 1 analytic accounts in relation to that conceptual attribute, and (iii) when saturation had been reached in terms of the concepts being meaningful and language having been fully tested and validated with the youngest and eldest potential users of the measure; the question of whether saturation had been reached was considered at the end of each batch of interviews (Arbuckle and Abetz-Webb, 2013; FDA, 2009).

## 3 Findings

Thirty-three in-depth Phase 1 interviews were conducted with CYP aged 11–15 (n = 19) and their parents (n = 14) (September 2019–November 2021) and fifteen semi-structured Phase 2 interviews were conducted with CYP (February–June 2022) across urban and rural areas of England. Ten interviews in Phase 2 were repeat interviews with informants from Phase 1. Informant characteristics for both phases are in Table 1. Phase 1 CYP interviews lasted between 16 and 60 min and parent interviews between 26 and 51 min. Phase 2 CYP interviews were between 11 and 51 min.

The decision to develop a distinct measure for the 11–15 age group came from the differences in capabilities expressed appearing to be influenced not just by age, but also by social context in terms of education, and so an early decision was made to draw the boundaries for the measures around the points at which this educational context changed. Data in relation to this measure were therefore analysed for the 11–15 group. For this 11-15 age group, after the analysis of Phase 1 data it was anticipated that nine attributes would be included in the measure; however, further analysis during Phase 2, suggested that two of these anticipated attributes (**Identity** and **Choice**) needed to be combined (**Identity and choice**), resulting in eight attributes for the final measure. No additional areas for consideration beyond these attributes were identified during Phase 2 interviews.

Data are presented below in relation to the final concepts and the attribute descriptors, in the order in which they appear on the final measure. Conceptual attributes are presented in bold, whilst final wording for the attributes is in bold and italics. Supporting evidence from CYP and parent interviews is included throughout. Quotations from the interviews are anonymised and any identifiable information removed to protect informants’ identities. Quotes from CYP are preceded with PC (indicating participant child), and quotes from parents with PA (indicating participant adult). All quotes are from Phase 1 unless specified. Hierarchical maps are not presented, but an example of a completed hierarchical map is provided in Appendix 1.

### 3.1 Conceptual attributes and wording for the measure

Through the interviews in Phases 1 and 2, eight conceptual attributes of capability wellbeing for CYP aged 11 to 15 were developed and are presented below.

#### 3.1.1 Fun and enjoyment: ‘Fun and enjoyment’

The importance of fun and enjoyment came out in early CYP interviews and was referred to by all 19 CYP. It was not, however, raised as important by parent informants. CYP talked about doing things that they found fun, such as spending time and doing activities with family and friends, and by engaging in hobbies and interests.

**PC024 (aged 12):** [discussing theme parks] “… they are really fun, and they’re really enjoyable, and it makes you feel happy …”

**PC111 (aged 14):** “[discussing piano] you watch videos of people and their fingers move so fast … I can’t move them that fast. I don’t play classical, but it’s still really cool and it’s really fun as well.”

The terminology used in the original conceptual attribute of **Fun and enjoyment** was well understood by informants in Phase 2 and was not changed for the final measure which remained as **Fun and enjoyment**. Wording was confirmed by Round 2 of the Phase 2 interviews.

#### 3.1.2 Learning and experiencing: ‘Learning and experiencing new things’

CYP informants suggested that they valued learning and experiencing new things, both in school and outside of school in terms of, for example, learning a new skill or visiting a new place. CYP suggested that they valued the process of understanding and doing things that they did not know or had not done before.

**PC101 (aged 12)**: “it’s always nice to learn new things and just to be able to do new things … like learning some new trick in maths or whatever …”

**PC035 (aged 15):** “Every year we go to [county in England] …. we do stuff that we don’t really do while we’re here. We get to go to the beach and we get to visit different places ….”

Some CYP also suggested that they valued change and the opportunities for personal growth that learning and experiencing new things allowed them.

**PC106 (aged 14):** “… if you don’t grow then you’re not doing anything, you’re not really furthering yourself …. Learning things and learning new skills and doing new things … just making yourself a better version of what you were yesterday.”

Interestingly, parent informants did not mention learning and experiencing new things as being important.

Again, the wording of learning and experiencing worked well with informants to express the **Learning and experiencing** conceptual attribute. It was found to be important to keep both terms in the descriptor, as learning was associated more with school whilst experiencing was seen more broadly, and adding ‘new things’ clarified the meaning to respondents (that is, ***Learning and experiencing new things***). Wording was confirmed by Round 2 of the Phase 2 interviews.

#### 3.1.3 Attachment: ‘Love and friendship’

All CYP suggested that they considered their relationships with family members to be important to them, with discussion focusing on the value that CYP placed on feeling close to family and happy in their company.

**PC032 (aged 12):** “my family is important to me because they are close to me …..”

CYP also talked about valuing the closeness and company of their friends, and the affection that they had towards family pets.

**PC104 (aged 14):** “We [friends] can go everywhere together, like to lunch, to lessons … You just know them really well …. I get to see them so much that they mean a lot to me.”

**PC109 (aged 14):** “they make you feel happy. And cats are very good for cuddles”

Parent informants also considered their child’s close connections with family members to be important, as parents suggested that it was important for CYP to feel loved.

**PA040:** “… know they’re loved and cared for even when it’s hard … know that they’ve got somewhere they can be … loved.”

Parents also raised the importance of CYP’s friendships, suggesting that socialising with peers was beneficial. Parents suggested that having meaningful relationships during childhood with family and friends would help their child to develop.

**PA105:** “She’s made some really good friendships … She’s had challenges with a couple of friends in the past but that all helps build … Some break down, some don’t work, but it’s a learning curve ….”

**PA100:** “to have enough love and enough friendship in your life to enjoy what’s going on … I think that’s really important.”

Phase 2 interviews explored terminology for the **Attachment** conceptual attribute, including both ‘love’ and ‘friendship’. It was seen as important to include friendship specifically in this measure given the growing importance of friendship groups at this age. Whether the terminology of ‘love’ was awkward for this age group was also explored, but was not found to be problematic. Wording of **Love and friendship** was established after Round 3 of the Phase 2 interviews.

#### 3.1.4 Emotional security and support: ‘Feeling emotionally supported and secure’

The importance of CYP feeling emotionally supported and secure came out early and strongly in both the CYP and parent interviews. This concept centred around the importance that CYP placed on feeling safe and not alone and having someone there for them for emotional support if they needed it, with this mostly applying to family, but also friends.

**PC104 (aged 14):** “my mum … is always there for me …. [she makes me feel] Happy, safe ….”

**PC042 (aged 12):** “ …. If I’m with one of my best friends I feel fine. If I’m with a person who I don’t really know, it’s a bit iffy.”

CYP further suggested that factors such as having a family home and a structure or routine contributed to their sense of emotional security.

**PC034 (aged 12):** “Well, this is my home … like I know it will always be there, like even though things change, my house will always be there. [I feel] Safe. Yes.”

**PC106 (aged 14):** “… in school there’s a bell, it tells you where you’ve got to go next … Without structure, unexpected things happen and scary things happen and … it’s uncomfortable, I don’t like it”

Parents also considered emotional security to be important to CYP and suggested that CYP’s sense of emotional security was centred on them having a stable family life and home; they also mentioned structure and routine as contributing to their child’s sense of emotional security.

**PA040:** “They just need stability … So have a house, to know they’ve always got somewhere they can go ….they can always come back to me …. they have a safe zone they can come to ….”

**PA102:** “… When he’s got a structure he feels safe … They feel secure, they know what they’re coming back home to.”

Similarly to the CYP, parents considered emotional support to be important to CYP’s wellbeing, in terms of having someone available to talk to and be there for them if they needed it. Parents suggested that this support would come from family, and increasingly friends, as their child got older.

**PA107:** “… she is going through puberty and periods and all that kind of stuff, she is happy to come and have a curl up and say ‘Can I have a little chat with you?’”

**PA037:** “… as you grow, you can share your experiences with your friends …. Someone who is going through the same experiences as you … who can understand you. I think that’s important.”

The early wording interviews for this **Emotional security and support** attribute and for the **Physical safety** attribute (see below) initially suggested that the two attributes might be indistinguishable from one another in wording of sufficient simplicity. Therefore wording was explored both for a combined attribute (‘safe and supported’) and for two separate attributes. Using the terms ‘emotional’ and ‘physical’ at the start of the wording for the two attributes did, however, seem to produce distinct responses and appeared to be understood by informants. Both separate and combined options were retained until the thinkaloud stage, where the combined option worked less well than the separate versions. Given that the attributes were distinct at the conceptual stage and it was feasible to separate them, the decision was taken to keep them separate. Wording of ***Feeling emotionally supported and secure*** was settled upon.

#### 3.1.5 Achievement: ‘Do well in the things that are important to me’

CYP and parents suggested that achievement was important to CYP’s quality of life. CYP suggested that they valued doing well at something and progressing in their chosen areas of interest, whether that be academically or in their hobbies.

**PC041 (aged 14):** “I want to get better [horse riding]. I kind of want to do competitions and show jumping.”

Parents suggested that achieving at school and in hobbies was important to their CYP, and parents thought that CYP realising and being praised for their achievements was key to their self-confidence.

**PA036:** “He got some awards at school last year, a lot of that was around the confidence … All of a sudden, he was speaking up whereas before he was always hiding in the background.”

The wording tried for the **Achievement** attribute was derived from how informants talked about this in Phase 1. Two wording choices were tried; ‘achieve the things that are important to me’ was rejected in favour of **Do well in the things that are important to me**, with this wording being established by the end of Round 2 as being most meaningful to informants.

#### 3.1.6 Identity and choice: ‘Free to be who I want to be and make the choices I want to make’

At the end of Phase one, two potential attributes were initially identified around **Freedom and making choices** and **Developing Identity**. Each of these is discussed below, before the process by which they were combined into a single attribute is discussed.

##### 3.1.6.1 Freedom and making choices

This concept focused on the value that CYP placed on being able to make choices, particularly being able to make choices about how they spend their time, including being able to have privacy and time alone.

**PC032 (aged 12):** “Choosing what I want to do … with my time …. sometimes I just don’t want to do something … Like look after people ….. And I don’t like walking the dog, that sort of thing.”

**PC041 (aged 14):** “… when I go out taking photos … it’s like you can just do what you want whilst you’re out there and you don’t have to get told what to do.”

Parent informants similarly considered their child’s freedom to be important, although this was more in the sense of them being allowed age-appropriate levels of independence.

**PA097:** “Now, she’ll go off and she’ll meet friends and go off for walks. Or we’ll drop her at the shopping centre, and she’ll go shopping and then we pick her up.”

Some parents suggested that it was important to give their child freedom now, to prepare them to be able to handle aspects of life independently, such as having responsibility over their own decisions and managing risk.

**PA105:** “ …. she’s got to have small little risks, hasn’t she? She’s got to be able to cope with those risks and manage those risks and, if something happens, know that she can deal with them ….”

##### 3.1.6.2 Free to be who I want to be

This concept relates to CYP’s expressions of their own identities and the type of person they were or wanted to be. CYP talked about their identities in relation to factors such as their cultural background, their religion, their appearance, and their hobbies/interests. A few informants suggested that they understood their own identity in relation to that of others, for example, in being a fan of a particular football team.

**PC109 (aged 14):** “… It feels like you’re like a new person, when you get different clothes … it’s nice experimenting with different styles of clothes, to see what you like ….I have had my hair different lengths … you can just go from long hair to shorter, to feel like a new person.”

Parents’ comments focused on the importance of CYP developing their identities in terms of them finding out who they are and where their passions and interests lie. Parents suggested that CYP knowing and being confident in their identities was important to their confidence.

**PA100:** “Just exploration about knowing yourself and knowing what you like and what you don’t like …. [being] happy with yourself …”

##### 3.1.6.3 Combined attribute: Identity and choice

Although separate wording was initially tried for these two preliminary attributes, following the first two rounds of wording interviews, it was becoming clear that informants were finding it difficult to separate these two attributes, seeing the two aspects as being inextricably aligned.

**PC038 (aged 15, Phase 2):** “I think that’s the same as being you, and making choices you want to make …. Because if you’re being you, then you would want to make those choices anyway.”

A decision was therefore made to explore wording for a combined **Identity and choice** attribute in Round 3, and a decision was taken at the end of this round to confirm a single attribute with wording of ***Free to be who I want to be and make the choices that I want to make***.

#### 3.1.7 Physical safety: ‘Being physically safe and protected’

Several CYP informants suggested that being physically safe and protected was important to them. CYP talked about their basic needs being met, such as having water, food, and a safe home. CYP suggested that these needs were often met by family members.

**PC043 (aged 12):** [My mum] “ …. gives me a home, she feeds me and everything …..if you don’t have a home or you didn’t get fed, you’d die or you’d be on the streets ….”

**PC113 (aged 11):** “I’ve got ‘food and water’ [on hierarchical map]. I wouldn’t live without them.”

Parents also talked about physical safety as important, which was centred on wanting their child to be free from any physical danger, including their safety outside of the house and online.

**PA107: “**… We’re on a little cul-de-sac, we’re very fortunate, and the area is not too bad, so they can play on the street, popping back every 15 minutes and all that sort of stuff.”

Parents also demonstrated concern for their child’s health, particularly future health, wanting this to be preserved for their CYP.

**PA031:** “You just worry these days, don’t you, about … self-harm and suicide and stuff.”

**PA054:** “Yes, so for me it’s about getting some healthy food into her as much as possible. I try and do as healthy food as possible for our evening meals”

As indicated above, the wording discussions for this **Physical safety** attribute were combined with those of the **Emotional security and support** attribute but the decision was made to retain them as separate attributes in response to the distinction between the two attributes perceived by CYP.

**PC111 (aged 15, phase 2):** “… you could have a roof over your head. You could have a door, windows. You could have food, you could have water … You could have everything that makes you feel safe but you’re not going to feel supported … It’s like even though you’re safe, you’re on your own.”

The final wording settled on for this element was **Being physically safe and protected** which matched the original intentions for this attribute.

#### 3.1.8 Aspiration: ‘Being able to think positively about my future’

Most of the discussion in the interviews focused on what was important to current wellbeing, but throughout the interviews with both CYP and parents, concerns about future wellbeing arose organically in a number of areas. There was particular discussion of the future in relation to the importance of education and academic achievement, with CYP emphasising the need to achieve now and in the future in order to get a good education and eventually a good job. Future achievement appeared to be valued (by both CYP and parents) on its own merit (that is, achieving good grades and getting a ‘good’ job) but also because some informants perceived academic achievement to be important to future freedom (that is, their education and job allowing them the freedom to do the things they want to do) and security in relation to finances.

**PC024 (aged 12):** “School is important to me because if I go to school, in later years, I can have a really good education and … about my future, and it can help me get jobs.”

**PA033:** “… to get GCSEs and A levels and have the options for the future ….you have more doors open to you if you have a degree … I’ll definitely encourage her to study, up to that level ….”

**PC034 (aged 12):** “I’d like a nice house … I just hope that I’ll be able to afford a house.”

Several CYP informants talked about future attachment, and the importance that they placed on having a family of their own in the future, or carrying forward existing friendships, with parents noting the importance of establishing important connections into adulthood.

**PC109 (aged 12):** “Getting married, having children. Yes …..Because I think, because I have grown up with a really nice family, it has made me think that I want that for my future.”

**PA110:** “ …. hopefully those friendships will last well into her adulthood. They’ll have memories together … It’s really lovely to have that.”

Discussions around future identity focused on the kind of people that the CYP wanted to be when they were older, which was sometimes discussed in relation to their future job choice. Parents also suggested that developing identities that they could be comfortable in now would be key to CYP having confidence in themselves into the future.

**PC106 (aged 14):** “I’d like to have furthered the cause of humanity in some way, be that building energy efficient cars with eco-friendly sustainable fuel cells or just helping a couple of people stay happy. I’d just like to be able to say that I have helped.”

**PA112:** “ …. being your own person and having your own strong identity that you carry through life. You should be proud of where you come from … ..”

Overall, there was evidence that CYP informants wanted to be able to aspire to particular futures. Rather than having present and future aspects of a number of attributes within the measure, these aspects of aspiration were taken together as an **Aspiration** attribute. Wording that was tried had both negative and positive connotations, around not worrying about the future, and thinking positively about the future. Although both wordings worked for the majority of informants, there was some preference expressed for the positive wording and that was taken forward as ***Thinking positively about my future***.

### 3.2 Presentation of the measure

A number of different presentational formats were explored with informants, including text only, and various visual images including smiley faces (from sad to happy), plants (from seed to fully grown plant), ladders (of varying length from short to long) and cups (from full to empty). The text only version was seen as ‘boring’, ‘a bit old’ and ‘not very engaging’ whilst the images were perceived as enhancing understanding.

**PC109 (aged 12)**: [text only version] “*I think it looks a little bit like an exam paper. (Laughter) It is not particularly encouraging …”*

**PC038 (aged 13):** “I think it looks better with the pictures …. for me, it just helps me picture it more and it’s all there for me … it’s easier to fill out, I would say.“

Ladders were almost universally disliked or felt to be uninformative, whereas plants were seen positively by some informants but negatively by others, who referred to them as ‘depressing’.

**PC103 (aged 12)**: “If I looked at- I mean it would probably make me think ’Oh look, someone chopped a ladder and then someone chopped a little bit off the ladder and then a little bit more’O … On first looking at it, it wouldn’t make me straight away see ’Oh, this is illustrating it ’”

**PC111 (aged 14)**: “it’s [plants] suggesting that if you don’t have any fun and enjoyment, you can’t grow.”

Smiley faces were liked by some informants and largely seen as clear, but some older CYP felt they were slightly childish. There was also concern that the facial expressions did not fully relate to the idea of capabilities, that is, that the happiness associated with a capability level might not be the same as the capability level itself.

**PC039 (aged 12):** “I think with ‘a little fun and enjoyment’, it wouldn’t be that sad. I think it’d just be like a straight face.”

In general, cups were most preferred by informants, who noted that they more clearly expressed the different levels of capabilities. They liked the use of colour in the image, and interestingly sometimes interpreted the image as batteries rather than cups.

**PC099 (aged 13): “**It is helpful because it, kind of, describes a bit more to people the different levels of how much it is.”

The decision was taken to use cups in the final version of the measure (see Appendix 2 for an example of the formatting of the final measure).

One other aspect explored in Phase 2 was the number of response levels to be included. Informants were divided in their preference for four or five levels, with some finding the wording too similar between attributes when five levels were used, but others preferring a ‘middle level’. Given this division, a pragmatic decision was taken to go with four levels, which would both simplify the measure and ease any associated valuation task (required to make the measure suitable for economic evaluation).

In terms of the top response levels for each attribute, in keeping with the ICECAP-A measure, a decision was made to keep the top level as “a lot” or “many”, instead of “all”. For some attributes for CYP, this is particularly appropriate, as for example, in relation to CYP being able to make all the choices that they want to make. However, during the Phase 2 interviews, the importance of being fully physically safe and protected became apparent, so a decision was made to change the top levels for the **Physical safety** and **Emotional security and** support attributes to “all”. The wording for the remaining levels is the same across all attributes - for the second from top level, “some” was chosen, with “a little” or “a few” chosen as the second from bottom level. The bottom level description is “no” or “none”.

## 4 Discussion

This work has generated the first capability measure for use with CYP in economic evaluation of health and care interventions. The measure was generated from in-depth qualitative data from CYP and their parents to generate conceptual attributes, and from CYP themselves to develop the associated wording and presentational format. Eight attributes were found to be important and were carried through to the measure: **Fun and enjoyment; Learning and experiencing; Attachment; Emotional security and support; Achievement; Identity and choice; Physical safety; Aspiration**. Although not directly comparable, studies focusing on subjective and psychological wellbeing in CYP in other high-income countries have also noted the importance of similar or related aspects of wellbeing, particularly **Attachment** (Fattore et al., 2009; Gonzàlez-Carrasco et al., 2019; Navarro et al., 2017; Vujčić et al., 2019), **Achievement** (Fattore et al., 2009; Gonzàlez-Carrasco et al., 2019), **Fun and Enjoyment** (Fattore et al., 2009; Vujčić et al., 2019), **Identity and choice** (Fattore et al., 2009), both **Physical safety** and **Emotional security and support** (Fattore et al., 2009) and **Aspiration** (Gonzàlez-Carrasco et al., 2019).

Compared with existing generic adult capability measures generated using participatory processes (for example, ICECAP-A (Al-Janabi et al., 2012) and ICECAP-O (Grewal et al., 2006)), there are two additional attributes with different foci. Both have a particularly critical relevance to child/young personhood, where CYP are growing and developing into the adult person they will become. The first is **Learning and experiencing** and the second is **Aspiration**. Interestingly, while parents spoke frequently about **Aspiration**, they did not discuss **Learning and experiencing**. For CYP though, this latter attribute appeared to be very important. Both these attributes fit well with existing literature on capability in CYP. **Learning and experiencing** speaks to the idea of evolving capabilities (Ballet et al., 2011) and ‘education’ also appears in the list of young people capabilities generated by Biggeri et al. (2006), while **Aspiration** fits well with Hart’s work on the capability to aspire (Hart, 2014). Compared with existing adult measures, the attributes that correspond to concepts of ‘stability’ (ICECAP-A (Al-Janabi et al., 2012))/’security’ (ICECAP-O (Grewal et al., 2006)) seem to be particularly important in this group. Both **Emotional security and support** and **Physical safety** were discussed extensively by both CYP and parents, and seemed to be distinct from one another in terms of the aspects covered. This perhaps relates to greater vulnerability of CYP than adults and the need therefore for protection from that vulnerability. This focus on safety and security also distinguishes **Emotional security and support** from the focus of the **Attachment** attribute on an intrinsic feeling of closeness to other beings, both human (family and friends) and animals. Among 11–15 year olds however, there is also increasing focus on **Identity and choice**, particularly around how they spend their time. This has some conceptual similarity with the capabilities of ‘religion and identity’ and ‘time autonomy’ derived by Biggeri et al. (2006).

A clear strength of the work is the richness of the interviews that were conducted with CYP and their parents, with each bringing different perspectives about the things that are important to CYP, enabling both perspectives to inform the understanding of the important aspects of capability wellbeing for CYP. Although consideration was given to including others’ perspectives (for example, teachers and health professionals), the approach used in other measures has been to focus on the population of interest; parents were included here mainly because of the issues around cognitive and, particularly, legal responsibility. The maximum variation sampling approach within the CYP and parent groups was largely successful in representing important characteristics, with the exception perhaps of a lack of parents from the most deprived backgrounds and a skew towards female participants in the Phase 2 interviews; recruitment from different minority ethnic groups also varied. Inevitably those who opted to take part in this research may be different from those who did not, however, attempts were made to ensure that those who opted to participate were compensated for their time, reducing some of the barriers to participation. Attempts to engage CYP using the hierarchical mapping activity were generally successful; during the in-person, pre-COVID phase of data collection, CYP particularly enjoyed using the polaroid camera to capture their image. Generating the map before recording began, meant that it was available as a prompt during the interview to stimulate discussion. This approach also facilitated CYP having a sense of control over the research (Barker and Weller, 2003; Punch, 2002) and potentially redressed some of the power imbalances between the adult researchers and CYP participants, as the CYP were able to talk freely through their maps. In Phase 2, the shift from testing wording directly to a thinkaloud format in later Phase 2 interviews was beneficial in exploring CYP’s understanding of the language used, and whether they were able to complete the measure in its final format. A further strength lies in the use of constant comparison for analysis. This rigorous process ensures that only like data are grouped together, and in concept elicitation, this facilitates the development of data into distinct categories to inform measure items. By challenging existing categories with new data, differences in the experiences and views of CYP are preserved and represented in the final measure (Strauss and Corbin, 1990). In all, our approach to data collection and analysis prioritised maintaining the voices of the CYP to inform measure attributes (for example, by including two attributes of **Fun and enjoyment** and **Learning and experiencing** that were not discussed by parents), aiming to ensure that the concepts contained are representative of what is important to the capabilities of future measure users. Inevitably, however, their voices are constrained by the context they are in, that of a high-income, twenty-first century setting.

The forced shift to online interviews due to COVID-19 had both advantages and disadvantages. The nature of online interviews meant that gaining rapport was more challenging; the task was still completed but lost some of the ‘fun’ elements. The presence of parents in interviews was also more difficult to manage online. Nevertheless, CYP still appeared comfortable (perhaps because of the pandemic-induced experience of conducting many interactions online), and spoke freely in online interviews; these interviews were no shorter than the face-to-face interviews, suggesting that it was still possible to capture the same richness of data. Whilst the shift to online interviews meant that it was possible to recruit from further afield, diversifying the sample in one way, it also means that those without access to digital resources would not have been able to take part post-COVID. One advantage of recruiting participants and collecting data within both time frames means that the findings are able to capture some informants’ perspectives pre-COVID and other informants’ perspectives during/post-COVID; there were no clear differences in what was valued across these time periods although the way in which the attributes were experienced showed differences.

This measure aims ultimately to provide an alternative approach to the measurement of benefits to CYP for economic evaluation of health and care interventions, one that will be better able to capture benefits associated with interventions targeted at improving the social determinants of health as well as service interventions. Further work is required, however, before that point is reached. The psychometric properties for the measure need to be tested, as well as its acceptability to CYP and their ability to complete it, with this testing needed across the general population and for specific groups, including different patient and care groups in the health and care context. If used in non-UK settings, consideration also needs to be given to the applicability of the age boundaries used for this measure, which in part reflect the point at which most UK CYP shift from primary to secondary education. Values for the measure also need to be obtained. Eliciting values for CYP populations is challenging but there has been some success in using best-worst scaling (Rogers et al., 2021), the approach already used by other capability measures (Mitchell et al., 2021). Further work is also needed in generating measures for other parts of the CYP element of the life course.

In conclusion, this work has generated information about the key capability wellbeing concepts that are important for children and young people of secondary school age. Potentially, the ICECAP-CYP:11-15 provides a valuable step forward in developing measures that can capture the benefits of health and care interventions, including those that focus on improving the social determinants of health for children and young people, and thus being able to assess the cost-effectiveness of these interventions.

## Supplementary Material

Multimedia component 1

Appendix

## Figures and Tables

**Table 1:** Characteristics of informants in Phases 1 and 2.

			Phase 1			Phase 2	
	CYP Characteristics		CYP	Associated Parents^a^		CYP Sample	
	Age (Years)						
	11		4	4		3	
	12		7^b^	6		5	
	13		2	1		2	
	14		4	4		3	
	15		2	1		2	
	Gender						
	Male		10	7		3	
	Female		9	9		12	
	Ethnicity						
	White British		13	12		10	
	Black		1	0		0	
	Asian		4	4		5	
	Other		1	0		0	
	Deprivation level^c^						
	1–3 (most deprived)		5	2		1	
	4–7		8	8		9	
	8–10 (least deprived)		6	6		5	
	Urban/rural						
	Urban		14	11		8	
	Rural		5	5		7	
	Health condition ^d^						
	Health condition		4	4			
	No health condition		15	12			
	Family						
	One parent		6	4		6	
	Two parents		13	12		9	

a Numbers of associated parents in the final column exceed the total number of parents in the sample because some parents were interviewed about multiple CYP.

b This number includes the perspective of one secondary parent who acted as a proxy for her 12-year-old daughter due to her having severe learning difficulties and being unable to participate in the study directly.

c Deprivation level determined using the English Index of Multiple Deprivation (IMD) measure, which ranks small areas in England from most deprived to least deprived in ten equal groups (with IMD decile 1 being most and decile 10 being least deprived).

d Not systematically collected in Phase 2.

## Data Availability

Data will be made available on request. Developing an ICECAP capability measure for children and young people aged 11-15 (Original data) (data.bris Rsesearch Data Depository)
