# Supplementary material for: The development of a capability wellbeing measure in economic evaluation for children and young people aged 11-15
Source: Soc Sci Med. Author manuscript; Available in PMC 2024 Nov 21. (PMC7616779; doi:10.1016/j.socscimed.2024.117311)
Supplement: Multimedia component 1 [file EMS199747-supplement-Multimedia_component_1.docx]

**The development of a capability wellbeing measure in economic evaluation for children and young people aged 11-15: Supplementary material**

**Appendix 1: Topic guide for children and young people**

| **Questions about the drawing task (attributes for measure):**   - Can you tell me about what you have drawn/written (*go through the items on A3 paper one by one*)?   ***Probe:***   - - Why is [item] important to you?   - Why do you like it? Why does it make you happy? What is it about it that is important to you?   - You have put [item A] as more important to you than [item B], why is that (continue for all items)? - Can you think of anything else that is important to you that you haven’t drawn or written on here? Has what is important to you changed since COVID-19? Is there anything that you did prior to Covid-19 that you aren’t able to do now?   **Questions about factors that impact quality of life:**   - Can you tell me about things that you don’t like? Is there anything in your life that makes you unhappy? - Is there anything that you think would make your life better than it is now?   **Questions about the future:**   - What would you like to do when you grow up (younger children)/What would you like to do in the future? - [Young people only] Where do you see yourself in the future? What will you be doing? What will you have? - Are there any other things that you would like to do/achieve? - [Young people only] Do you think the things that are important to you have changed over the years? |
| --- |

**Appendix 2: Interview topic guide for parents/guardians**

| **Questions about child’s quality of life (wellbeing):**   - Can you start by telling me a bit about how your child spends their time (typical days, evenings, weekends)? - Can you tell me about how you spend time as a family? What do you like to do? - What things do you consider to be important to your child’s quality of life?   - What things do you consider to be important to your child’s happiness?     - In terms of what you want them to do?     - In terms of how you want them to feel?   - *Probe each:* What is it about these things that makes them important?   - Which of these things do you think is most important? How do they compare? - What do you think your child considers to be important in their life? Why do they consider these things to be important? - Is there anything that you think could improve your child’s quality of life?   - Anything that you are not so happy with?   - Things that you would like more/less of in your child’s life?   - *Probe: why do you think these factors make your child’s quality of life poor/will improve your child’s quality of life?* - [Other parents have said ‘x is important’, what do you think?] - Do you think that what is important to you/your child has changed since Covid-19 (probe whether this is a permanent or temporary change)?   **Questions about child/young person’s future (well-becoming):**   - What would you like for your child in the future? - What things do you consider important to your child’s future happiness? - Do you think what is important to your child now is the same as when they were [x] age? |
| --- |

Appendix 3: Example topic guide for Phase 2 (version from round 3)

**Children and Young People Quality of Life Study**

**Topic guide for secondary aged CYP**

**Prior to interview:**

- [Re]introduce self to child/young person
- If CYP has taken part in phase 1, remind them of this and what their previous involvement was. If this is CYP’s first involvement, explain what happened in phase 1. Inform all participants of the attributes that emerged as important in phase 1 (see below question on coverage of attributes).
- Make sure child/young person has completed written assent form & parent consent form
- Remind child/young person that they do not have to answer every question if they don’t want to
- Tell them they can withdraw from the research at any time
- Check that the child/young person is happy to have their interview recorded. If yes, switch on recording!
- **Ask for verbal consent on the tape**

**Study summary:** Explain to CYP that we want to find out how we can turn those things that are important to you and other children and young people into a questionnaire that everyone your age can complete. And we need your help with how we word the questions and how the questionnaire looks.

**Questions on wording:**

I am going to ask you some questions about some words and I’m interested in knowing what you think those words mean and how you understand them. There are no wrong answers, I am just interested in talking to you about what you think about when I say a word. Ask questions about wording from at least four of the sections below (1-8).

1. **Able to have fun and enjoyment**

- When I say to you *“have fun and enjoyment”,* what do you think about?

1. **Feeling supported and safe**

- When I say to you *“feel supported and safe”*, what do you think about? What makes you feel like this?
- What about if I just say to you *“feel safe”, what would be missing (when compared to feel safe and supported)?*
- What about if I just say to you *“feel supported”, what would be missing (when compared to feel safe and supported)?*
- What if I said, *“emotionally supported and secure”, what would that mean to you?*
- What if I said, “*physically safe and protected*”, what would that mean to you?

1. **Able to think positively about my future**

- What if I said to you *“I am to think positively about my future”,* **what sort of things does that make you think about?**
- What if I said, “*I am able to think about my future without concern”?* **What sort of things does that make you think about?**
- What if I said, *“I am able to think about my future without worry”?* What sort of things does that make you think about?
- Which of these options do you prefer?

1. **Learn and doing new things**

- When I say to you *“learning and experiencing new things”*, what do you think about?

1. **Love and friendship**

- When I say to you *“love and friendship”?* What do you think about?
- What about when I say to you “*love and friendship from those who care about me”*, what does that make you think about? How is that different to the first wording?
- What about if I say, *“love and friendship from those who are close to me”?* Is that the same or different?

1. **Do well in the things that are important to me**

- What does “*do well in the things that are important to me”* mean to you?

1. **Free to be who I want to be and able to make the choices I want to make**

- If I said to you, “*I can be me*”, what does that mean to you?
- What if I said, *“free to be who I want to be”,* what does that mean to you?
- What about if I said, “I can make the choices I want to make”, what does that mean to you?
- What about if I said, “*free to be who I want to be and make the choices I want to make”*, what does that mean to you?
- What about if I said, “*I can be me and make my own choices?”* What does that mean to you?
- What about if I said, *“I can be myself and make my own choices?”*

**Questions on coverage of attributes**

- The things identified as being most important to children and young people’s happiness or wellbeing through interviews with CYP were: *fun and enjoyment, feeling safe and supported, able to think positively about my future, learning and experiencing new things, love and friendship, do well in the things that are important to me, free to be who I want to be and make the choices I want to make*.
- What do you think to that list?
- Do you think that covers all of the main/overall things that are important to you?
- Can you think of anything that might be missing from that list? Is there anything that you would like to add to it?
- Do any of those stand out as being more or less important to you?

**Questions on questionnaire formatting**

I am going to ask you some questions about some pictures that we might use in the questionnaire.

- [Show CYP word only version] what do you think to how the question looks? Would you find that easy to fill in? How would you answer? Why?
- [Show CYP particular image] what does this picture mean to you? What does it make you think about? What do you think the picture is saying?
- [Remind CYP of different versions of the questionnaire (i.e., words only, pictures and words)] Do you like any of these options better than others?
