# Supplementary material for: The development of a capability wellbeing measure in economic evaluation for children and young people aged 11-15
Source: Soc Sci Med. Author manuscript; Available in PMC 2024 Nov 21. (PMC7616779; doi:10.1016/j.socscimed.2024.117311)
Supplement: Appendix [file EMS199747-supplement-Appendix.pdf]

**Appendix 1. Example of hierarchical map**

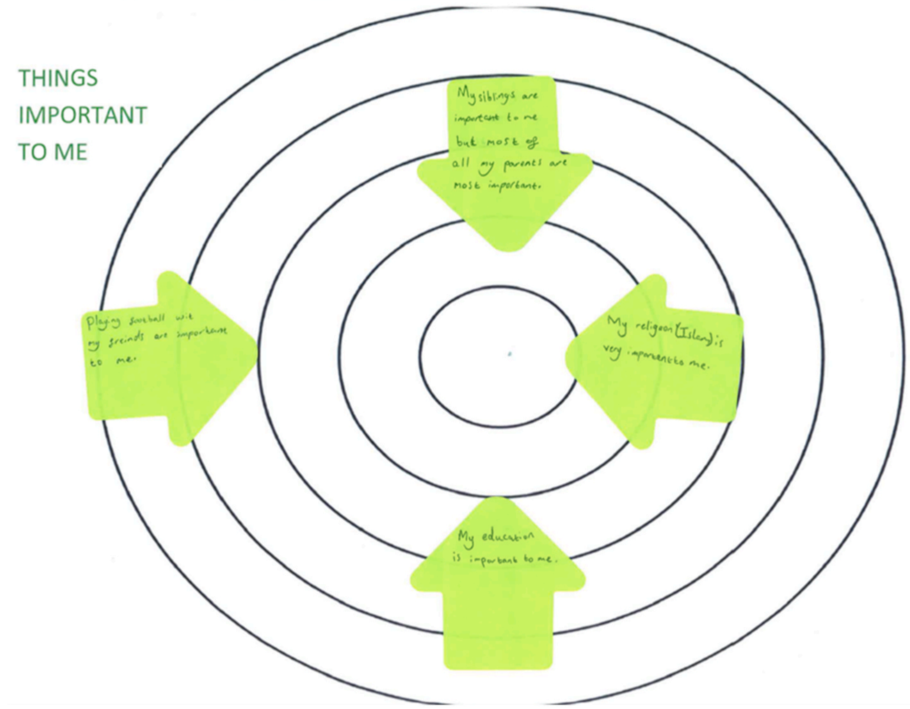


**Appendix 2. Excerpt showing final formatting of ICECAP-CYP:11–15 measure**

**ABOUT YOUR LIFE AT THE MOMENT**

Please put a tick (✓) in ONE box for EACH question below to show which option best describes your life at the moment.

**1. Have fun and enjoyment**

| I can have **a lot of** fun and enjoyment | I can have **some** fun and enjoyment | I can have **a little** fun and enjoyment | I can have **no** fun and enjoyment |
|---|---|---|---|
| | | | |
| ☐ | ☐ | ☐ | ☐ |

**Appendix A. Supplementary data**

Supplementary data to this article can be found online at https://doi.org/10.1016/j.socscimed.2024.117311.
